# Trends in the registration of anxiety in Belgian primary care from 2000 to 2021: a registry-based study

**DOI:** 10.3399/BJGP.2022.0196

**Published:** 2023-03

**Authors:** Simon Gabriël Beerten, Kristien Coteur, Pavlos Mamouris, Marc Van Nuland, Gijs Van Pottelbergh, Lidia Casas, Bert Vaes

**Affiliations:** Department of Public Health and Primary Care, KU Leuven, Leuven.; Department of Public Health and Primary Care, KU Leuven, Leuven.; Department of Public Health and Primary Care, KU Leuven, Leuven.; Department of Public Health and Primary Care, KU Leuven, Leuven.; Department of Public Health and Primary Care, KU Leuven, Leuven.; Social Epidemiology and Health Policy, Department of Family Medicine and Population Health, and Institute for Environment and Sustainable Development, University of Antwerp, Antwerp, Belgium.; Department of Public Health and Primary Care, KU Leuven, Leuven.

**Keywords:** anxiety, Belgium, epidemiology, general practice, primary care, comorbidity

## Abstract

**Background:**

Anxiety is frequently encountered in general practice, but figures regarding prevalence and incidence in this healthcare setting remain scarce.

**Aim:**

To provide insight about the trends in prevalence and incidence of anxiety in Belgian general practice, as well as the comorbidities and treatment of anxiety in this context.

**Design and setting:**

Retrospective cohort study using the INTEGO morbidity registration network, with clinical data from over 600 000 patients in Flanders, Belgium.

**Method:**

Trends in age-standardised prevalence and incidence of anxiety from 2000 to 2021 as well as prescriptions in patients with prevalent anxiety were analysed with joinpoint regression. Comorbidity profiles were analysed using the Cochran–Armitage test and the Jonckheere–Terpstra test.

**Results:**

During the 22-year study period, 8451 unique patients with anxiety were identified. The prevalence of anxiety diagnoses rose significantly during this period, from 1.1% in 2000 to 4.8% in 2021. The overall incidence rate rose from 1.1/1000 patient–years (PY) in 2000 to 9.9/1000 PY in 2021. The average chronic disease count per patient increased significantly during the study period, from 1.5 to 2.3 chronic conditions. The most frequent comorbidities in patients with anxiety in 2017–2021 were malignancy (20.1%), hypertension (18.2%), and irritable bowel syndrome (13.5%). The proportion of patients treated with psychoactive medication rose from 25.7% to almost 40% over the study period.

**Conclusion:**

A significantly increasing prevalence and incidence of physician-registered anxiety was found in the study. Patients with anxiety tend to become more complex, with more comorbidities. Treatment for anxiety in Belgian primary care is very dependent on medication.

## INTRODUCTION

Anxiety disorders are common,^1^ with a global prevalence of 4.1% in 2019, higher in females.^2^^,^^3^ In Belgium, the estimated prevalence in 2019 was 5.6% (6.5% for females and 4.7% for males).^4^ These results were derived from surveys (face-to-face interviews using the World Mental Health-Composite International Diagnostic Interview questionnaire) in the general population.^5^

In primary care, the most commonly diagnosed anxiety disorder is generalised anxiety disorder (GAD).^6^ The 2018 Belgian Health Interview Survey put the prevalence of GAD at 11.2%.^7^ It was a population-level self-report survey and the seven-item Generalized Anxiety Disorder scale was used.^8^ An important problem with data for mental health disorders is the discrepancy between diagnostic data in electronic medical records and self-reported data.^9^ Regarding trends in the epidemiology of anxiety there are many sources with contradictory findings. For instance, in a population-level analysis by Booth *et al,* anxiety tended to increase more over time in specific populations (for example, students).^10^ For most of these studies surveys such as the State-Trait Anxiety Inventory were used instead of clinical data. Furthermore, there are generally high rates of comorbidity between depression and anxiety.^11^^,^^12^ This has raised doubts as to whether these disorders constitute separate clinical entities.^13^ Some studies found common overlap for anxiety and mood disorders in primary care.^14^^,^^15^

As a result of the paucity of data surrounding trends in anxiety in primary care, this study sought to examine the registration of anxiety, its comorbidities, and medication prescription over a 22-year period in primary care in Flanders, the northern region of Belgium.

## METHOD

### Study design and data collection

This study used the INTEGO database, a morbidity registration network in Flemish primary care.^16^ It started in 1994 and contains medical information on over 600 000 patients. Primary care practices apply for inclusion in this registry, which is a continuous process. Registration performance of these practices is compared with that of other practices. Only those practices with optimal performance are included in this study. All diagnoses and new drug prescriptions are registered using codes.

In Belgium, patients are able to visit any GP they prefer. In INTEGO, their data are coded with a unique patient ID to avoid double-counting when they visit multiple GPs in the network. Currently, there are 104 practices in INTEGO. The patients of those practices are representative for the Flemish population in terms of age and sex.^16^ The study period ran from 1 January 2000 to 31 December 2021. Only practices with at least 80% coded diagnoses were included in the study (86 practices) to minimise intrapractice variation.

**Table:** How this fits in

Figures on anxiety in general practice are scarce. This study shows increasing incidence and prevalence of physician-registered anxiety. Patients with anxiety had an increasing number of comorbidities over time. Treatment of anxiety in this setting seems very dependent on medication, particularly selective serotonin reuptake inhibitors and anxiolytics.

The denominator used in this study is the yearly contact group (YCG),^16^^,^^17^ which contains all the patients who visited a practice at least once in a given year. Given that Belgian GPs do not have fixed patient lists, using the YCG as a denominator is therefore the most realistic approach.^17^ Included patients were aged ≥15 years.

### Anxiety, comorbidities, and treatment

Diagnoses in INTEGO are coded using the International Classification of Primary Care 2 (ICPC-2). To identify patients with anxiety the ICPC-2 code P74 ‘Anxiety disorder/anxiety state’ was used. In a sensitivity analysis the symptom code P01 ‘Feeling anxious/nervous/tense’ was added for comparison (see Supplementary Appendix S1, Table S1, and Figure S1). In line with the view that anxiety disorders are considered chronic,^18^ a ‘case’ was considered prevalent when the diagnosis of P74 was made at any point before the year of analysis during the study period. At the start of the study period (2000), a case was considered prevalent if the diagnosis had been made in 1999 or before. Similarly, a case was considered incident in a certain year if in that year the diagnosis was made for the first time ever. Incident cases were thus eligible to become prevalent cases in the next year. It was not possible to tell the severity of the disorder as this was not coded in the electronic health record.

For the comorbidities at the moment of diagnosis in incident cases (that is, present the same day as the first anxiety diagnosis) a precompiled list of chronic diseases was used, as in a previous study by the same author group.^19^ Chronic kidney disease was assessed by the closest creatinine measurements in the 2 years preceding or following a P74 diagnosis.

To define treatment, the Anatomic Therapeutic Classification codes that are available in the INTEGO databases were used. Psychoactive medication was defined as: N05A (antipsychotics), N05B (anxiolytics), N05C (hypnotics/sedatives), and N06A (antidepressants). The full list can be found in Supplementary Appendix S2. Medication prescriptions were assessed for patients with anxiety (although not necessarily prescriptions for anxiety) who received prescriptions on a) only one or b) ≥3 separate occasions a year, the latter serving as an approximation for chronic use. Interventional trials aimed at de-prescribing benzodiazepines define ≥3 months as long-term use.^20^

### Data analysis

Age-standardised prevalence proportions (/100 patients) and incidence rates (/1000 patient–years [PY]) were calculated. For age-standardisation, the Flemish population in Belgium in 2000 was used as the reference population. The following age groups were used: 15–29, 30–44, 45–59, 60–74, and ≥75 years. To analyse the trend in those age-standardised rates, joinpoint regression was used.^21^ From this model, the annual percentage change (APC) and the average annual percentage change (AAPC) are derived. The APC is calculated for each significant trend from a piecewise log-linear model on the logarithm of the age-standardised rate versus the year. The AAPC represents the average of APC estimates per significant trend weighted by the corresponding number of years in the trend.^21^

Based on the consensus in the article of Nielen *et al*,^22^ a sensitivity analysis was also performed using a contact-free interval of 1 year for the prevalence calculation (see Supplementary Appendix S3, Table S2, and Figure S2). The contact-free interval is defined as the period in which repeated visits with the same registered problem are considered to be part of the same episode of care.^22^

Trends in comorbidities in the incident anxiety case group were analysed using the Cochran–Armitage and the Jonckheere– Terpstra tests, whereas medication prescriptions in the prevalent anxiety case group was analysed using joinpoint regression. Medication prescriptions in the incident case group and by age group in the prevalent case group can be found in Supplementary Appendix S4 and Figures S3–S5.

Most statistical analysis was done using R (version 4.0.3). Joinpoint regression was done using the Joinpoint Regression Program (version 4.9.0.1) of the US National Cancer Institute.^21^

## RESULTS

### Trends in the prevalence and incidence of anxiety

**Figure 1.**
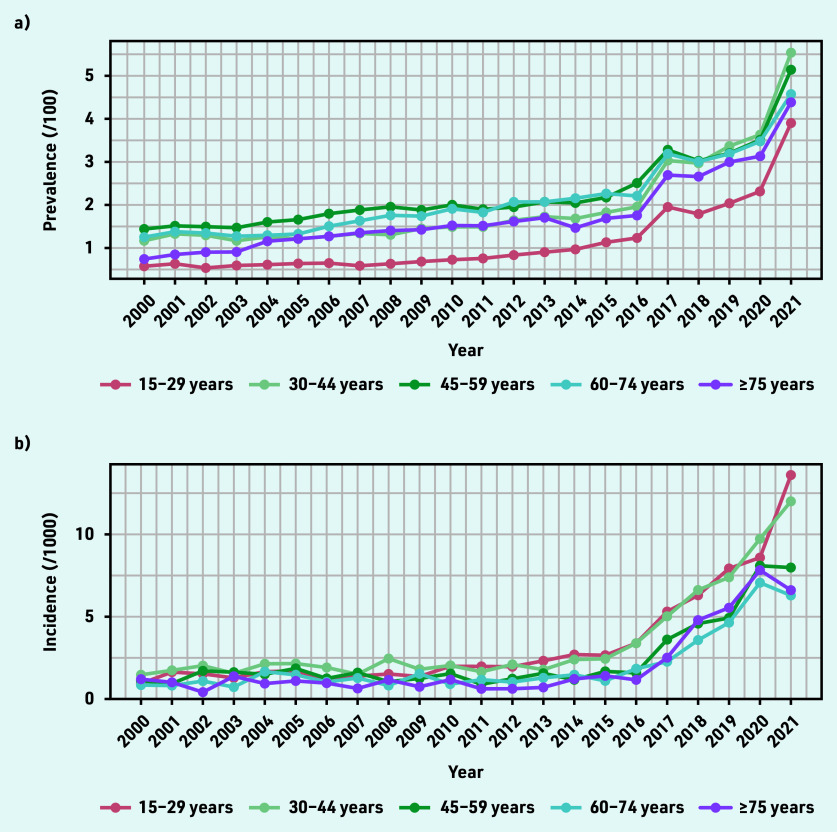
*a) Age-stratified prevalence and b) incidence of anxiety (2000–2021).*

During the study period, 8451 unique patients were identified with anxiety, using a chronic disease assumption. The prevalence of anxiety rose significantly over this period, from 1.1% in 2000 to 4.8% in 2021 (AAPC 6.8%, 95% confidence interval [CI] = 5.3 to 8.4). The APC from 2000 to 2015 was 3.4% (95% CI = 1.8 to 5.1), with a steeper rise of 15.8% (95% CI = 11.6 to 20.2) from 2015 to 2021 (Table 1 and Figure 1).

**Table 1.** Trends in the age-standardised prevalence and incidence of anxiety in Flanders, Belgium (2000 2021)

**Variable**	**Summary**			**Trend 1**		**Trend 2**	

	**Year 2000**	**Year 2021**	**AAPC (95% CI)**	**Years**	**APC (95% CI)**	**Years**	**APC (95% CI)**
**Prevalence (/100)**							
Total	1.07	4.82	6.8 (5.3 to 8.4)	2000–2015	3.4 (1.8 to 5.1)	2015–2021	15.8 (11.6 to 20.2)
**Sex**							
Female	1.38	5.80	6.5 (5.1 to 8.0)	2000–2015	3.4 (1.9 to 4.9)	2015–2021	14.9 (10.9 to 19.0)
Male	0.73	3.57	7.0 (5.3 to 8.6)	2000–2014	3.0 (1.1 to 5.0)	2014–2021	15.3 (11.5 to 19.2)
**Age, years**							
15–29	0.56	3.92	9.1 (6.8 to 11.5)	2000–2014	3.8 (1.0 to 6.7)	2014–2021	20.5 (15.3 to 25.9)
30–44	1.17	5.55	7.3 (5.7 to 8.9)	2000–2015	2.9 (1.2 to 4.6)	2015–2021	19.0 (14.3 to 24.0)
45–59	1.44	5.15	5.4 (3.9 to 7.0)	2000–2015	2.6 (1.0 to 4.2)	2015–2021	12.7 (8.3 to 17.3)
60–74	1.25	4.58	6.2 (5.0 to 7.5)	2000–2016	4.4 (3.2 to 5.6)	2016–2021	12.3 (8.3 to 16.5)
≥75	0.73	4.39	7.5 (5.9 to 9.1)	2000–2016	4.9 (3.2 to 6.5)	2016–2021	16.4 (11.4 to 21.6)

**Incidence rate (/1000 PY)**							
Total	1.14	9.94	10.3 (8.6 to 12.0)	2000–2014	1.8 (−0.2 to 3.9)	2014–2021	29.4 (25.6 to 33.3)
**Sex**							
Female	1.47	12.43	9.7 (7.8 to 11.6)	2000–2014	0.9 (−1.3 to 3.2)	2014–2021	29.7 (25.5 to 34.0)
Male	1.40	6.76	8.0 (6.1 to 10.0)	2000–2011	−2.7 (−5.8 to 0.6)	2011–2021	21.2 (18.8 to 23.5)
**Age, years**							
15–29	0.96	13.58	11.4 (9.0 to 14.0)	2000–2013	3.6 (0.3 to 7.0)	2013–2021	25.5 (21.2 to 30.0)
30–44	1.48	12.00	9.4 (7.5 to 11.3)	2000–2013	0.3 (−2.1 to 2.8)	2013–2021	26.0 (22.4 to 29.8)
45–59	1.11	7.95	9.3 (5.8 to 12.8)	2000–2014	−0.7 (−4.6 to 3.4)	2014–2021	32.2 (24.0 to 40.9)
60–74	0.87	6.30	9.7 (5.7 to 13.9)	2000–2014	0.9 (−3.9 to 5.8)	2014–2021	29.9 (20.8 to 39.7)
≥75	1.17	6.60	10.1 (4.4 to 16.1)	2000–2012	−3.4 (−11.3 to 5.2)	2012–2021	31.2 (22.2 to 40.8)

*AAPC = average annual percent change. APC = annual percent change. PY = patient–years.*

The same trend can be seen for the incidence of anxiety. The overall incidence rate in 2000 was 1.1/1000 PY and rose to 9.9/1000 PY in 2021 (AAPC 10.3%, 95% CI = 8.6 to 12.0). The largest increase in incidence was observed from 2014 to 2021, with an APC of 29.4% (95% CI = 25.6 to 33.3) (Table 1 and Figure 1).

The trends in prevalence and incidence for both sexes were similar, with the prevalence in females being almost twice that of males (Table 1). Concurrent increases in prevalence proportions were seen in all age groups, with the highest prevalence figures in the middle-aged groups (aged 30–59 years). The highest increase was in those aged 30–44 years. Incidence rates also increased substantially in all age groups, with the youngest age group contributing the most (Table 1).

When taking P74 and P01 together, both the incidence and prevalence increased (because of the now increased number of cases of patients with anxiety available), but the trends were largely conserved (see Supplementary Appendix S1, Table S1, and Figure S1).

### Trends in anxiety comorbidities

The relationship between anxiety and various groups of comorbidities are outlined in Table 2. The mean age at diagnosis showed a significantly positive trend, going from 44.1 to 45.4 years during the study period.

Psychological diagnoses associated significantly with anxiety disorder were depression and personality disorder. Various other somatic conditions were also associated with anxiety, for instance, cardiovascular, metabolic, and pulmonary disease. The average chronic disease count per patient increased significantly during the study period from 1.5 to 2.3 (Table 2).

**Table 2.** Trends in comorbidities in patients with anxiety in Flanders, Belgium (2000 2021)

**Variable**	**2000–2003**	**2004–2007**	**2008–2011**	**2012–2016**	**2017–2021**	**Trend test, *P-*value^a^**
**Patients with anxiety, *n***	473	659	694	968	5657	—

**Age, years, mean (SD)**	44.1 (17.4)	44.8 (17.4)	42.7 (18.2)	41.7 (18.0)	45.4 (19.6)	0.0006

**Sex, female, *n* (%)**	300 (63.4)	446 (67.7)	474 (68.3)	602 (62.2)	3783 (66.9)	0.4447

**Prevalence of comorbidities**						
Chronic disease count per patient, mean (SD)	1.5 (1.8)	1.7 (1.9)	1.7 (2.0)	2.1 (2.4)	2.3 (2.6)	**0.0002**
**Comorbidity and ICPC-2 code, *n* (%)**						
Depressive disorder (P76)	40 (8.5)	37 (5.6)	33 (4.8)	71 (7.3)	556 (9.8)	**<0.0001**
Alcohol misuse (P15–16)	9 (1.9)	25 (3.8)	16 (2.3)	49 (5.1)	184 (3.3)	0.4162
Dementia (P70)	3 (0.6)	5 (0.8)	2 (0.3)	11 (1.1)	60 (1.1)	0.1071
Schizophrenia (P72)	4 (0.8)	4 (0.6)	5 (0.7)	6 (0.6)	53 (0.9)	0.3822
Suicide/suicide attempt (P77)	1 (0.2)	1 (0.2)	0 (0)	5 (0.5)	18 (0.3)	0.2938
Phobia/compulsive disorder (P79)	14 (3.0)	19 (2.9)	23 (3.3)	43 (4.4)	166 (2.9)	0.7287
Personality disorder (P80)	3 (0.6)	7 (1.1)	9 (1.3)	32 (3.3)	243 (4.3)	**<0.0001**
Anorexia nervosa/bulimia (P86)	3 (0.6)	1 (0.2)	3 (0.4)	1 (0.1)	8 (0.1)	0
Substance misuse (P18–19)	2 (0.4)	4 (0.6)	10 (1.4)	15 (1.5)	64 (1.1)	0.2067
Atrial fibrillation/flutter (K78)	8 (1.7)	11 (1.7)	12 (1.7)	17 (1.8)	190 (3.4)	**0.0002**
Hypertension (K86–87)	57 (12.1)	100 (15.2)	99 (14.3)	135 (13.9)	1032 (18.2)	**<0.0001**
Heart failure (K77)	2 (0.4)	3 (0.5)	5 (0.7)	9 (0.9)	95 (1.7)	**0.0002**
Atherosclerosis (K92)	9 (1.9)	11 (1.7)	9 (1.3)	25 (2.6)	102 (1.8)	0.8476
Ischaemic heart disease (K74–76)	14 (3.0)	27 (4.1)	29 (4.2)	34 (3.5)	183 (3.2)	0.3879
Diabetes mellitus (T89–90)	16 (3.4)	29 (4.4)	51 (7.3)	77 (8.0)	380 (6.7)	**0.0054**
Hypothyroidism (T86)	8 (1.7)	12 (1.8)	5 (0.7)	22 (2.3)	216 (3.8)	**<0.0001**
Hyperthyroidism (T85)	4 (0.8)	4 (0.6)	0 (0)	1 (0.1)	20 (0.4)	0.2456
Irritable bowel syndrome (D01 and D93)	66 (14.0)	96 (14.6)	101 (14.6)	150 (15.5)	761 (13.5)	0.3133
Asthma (R96)	40 (8.5)	64 (9.7)	81 (11.7)	138 (14.3)	746 (13.2)	**0.0003**
COPD (R95)	10 (2.1)	22 (3.3)	23 (3.3)	32 (3.3)	227 (4.0)	**0.0263**
Osteoarthritis (L89–91)	57 (12.1)	87 (13.2)	69 (9.9)	114 (11.8)	613 (10.8)	0.1695
Cerebrovascular disease (K90–91)	7 (1.5)	9 (1.4)	12 (1.7)	20 (2.1)	143 (2.5)	**0.0127**
Malignancy^b^	18 (3.8)	36 (5.5)	52 (7.5)	94 (9.7)	1137 (20.1)	**<0.0001**
Chronic kidney disease^c^	4 (0.8)	6 (0.9)	3 (0.4)	8 (0.8)	72 (1.3)	0.0807

a *Bold indicates P-values for trend <0.05.*

b *Codes: A79, B74, D74–77, N74, R84–85, S77, T71, U75–77, W72, X75–77, Y77–78.*

c *No codes were used for chronic kidney disease due to issues with reliability; instead, the authors calculated glomerular filtration rate for each patient and decided who had chronic kidney disease based on these values. COPD = chronic obstructive pulmonary disease. ICPC-2 = International Classification of Primary Care 2. SD = standard deviation.*

### Trends in medication prescriptions in patients with anxiety

**Figure 2.**
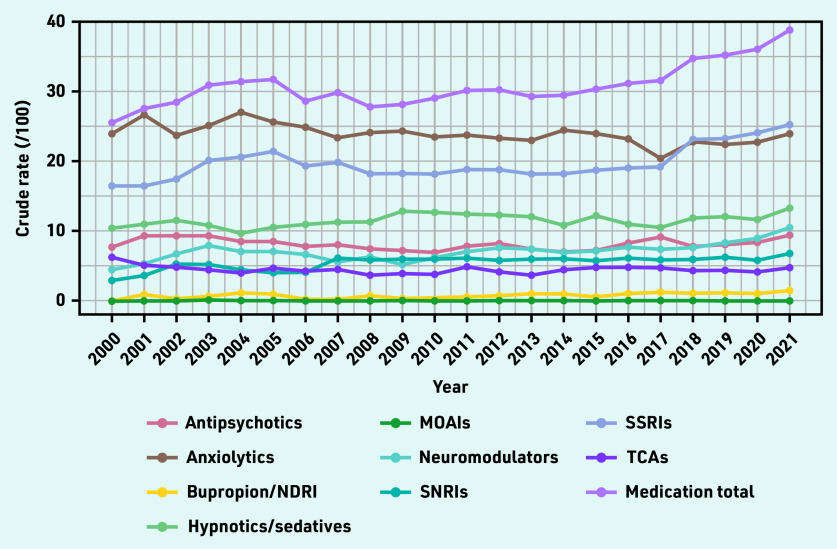
*Psychoactive medication prescriptions in prevalent anxiety case group (2000–2021). MAOI = monoamine oxidase inhibitor. NDRI = noradrenaline–dopamine reuptake inhibitor. SNRI = serotonin–noradrenaline reuptake inhibitor. SSRI = selective serotonin reuptake inhibitor. TCA = tricyclic antidepressant.*

In the prevalent anxiety case group, the proportion of patients treated with psychoactive medication rose from 25.7% to almost 40% (Figure 2). The increase was statistically significant for the period 2014 to 2021, resulting in an APC of 4.0% (95% CI = 3.0 to 5.1) (data not shown). In the incident anxiety case group, the proportion of patients treated with medication rose from 18.6% to 31.2%. This increase was also statistically significant with an AAPC of 2.0% (95% CI = 0.6 to 3.5).

When looking at patients receiving prescriptions once per year, the proportion was 3.8% in 2000, decreasing to 1.7% in 2021, with a significant AAPC of −4.4% (95% CI = −6.8 to −2.0). However, the proportion of patients receiving prescriptions ≥3 times per year went from 18.6% to 30.7% (AAPC 2.3%, 95% CI = 1.2 to 3.3) (data not shown).

This increase in the overall data is predominantly caused by a larger proportion of the study population being treated with selective serotonin reuptake inhibitors (SSRIs; APC 5.3% from 2014 to 2021, 95% CI = 3.8 to 6.7) whereas the use of anxiolytics tended to fall significantly, but not greatly (AAPC −0.5%, 95% CI = −0.8 to −0.2). Other psychoactive drugs were prescribed much less and their use stagnates or declines slightly, with the notable exception of neuromodulators (such as trazodone): their use more than doubled over the study period (from 4.5% to 10.5%, AAPC 4.0%, 95% CI = 1.2 to 6.9); fluctuating at the beginning of the study period (significant APCs of 20% for 2000–2003 [95% CI = 6.2 to 35.5] and −5.5% [95% CI = −9.3 to −1.6] for 2003 to 2009), their prescription rate steeply increased from 2018 to 2021 (APC 11.3%, 95% CI = 7.4 to 15.3) (data not shown).

**Figure 3.**
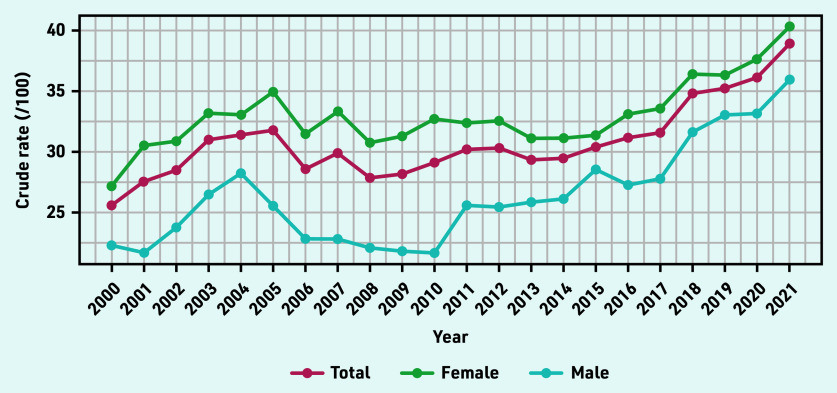
*Total prescription volume in prevalent anxiety case group (2000–2021).*

Proportionally, females with anxiety were prescribed more psychoactive medication than males (two-tailed *t*-test: *P*<0.0001; Figure 3). In both sexes, medication use increased over the study period.

When looking at specific age groups, younger age groups were prescribed more medication in the incident case group, whereas older groups were prescribed more in the prevalent case group (see Supplementary Appendix S4 and Figures S3–S5).

## DISCUSSION

### Summary

This study found that from 2000 to 2021 the prevalence and incidence of anxiety in Flemish primary care increased steeply, but mostly over the past 5 years. The younger age groups seem to be responsible for most of this increase. The number of comorbidities in patients with anxiety rose significantly during the study period. Medication prescription among patients with anxiety also increased, most notably for SSRIs.

### Strengths and limitations

The major strength of this study is the inclusion of longitudinal data, reflecting the registration trends of anxiety as they developed during the 22-year study period. Access to prescription and comorbidity data allowed for a balanced and multifaceted view on anxiety as it presents to GPs in Flanders. Given the paucity of real-world data on anxiety in primary care, the authors believe the current study is a valuable addition to the evidence. The study also comes with some limitations. The denominator of the study population can vary because of the fact that patients in Belgium are not registered with a particular GP. Although only 40% of the population go to see one GP only, the Usual Provider Continuity Index still remains acceptable (>75% of consultations with a specific GP for >65% of the Belgian population).^23^ Data for patients going to a GP outside of the INTEGO network, however, are not included.

The YCG also does not capture the whole practice population, but only the patients who visit at least once a year. It is, however, the most realistic approach in Belgium. The authors do not know whether GPs over- or underdiagnosed anxiety in their patients, and what clinical tools they used to arrive at their diagnosis. Also, there might exist regional variations in coding practice (for example, some practices are more likely to code anxiety as a symptom and not a diagnosis), something which this study did not specifically investigate. Diagnoses detailed as free text were not included in this study.

Many patients with anxiety problems might not go to their GP, leading to an underestimation of the real disease burden. The effect of the COVID-19 pandemic on anxiety was not specifically evaluated. Medication could have been prescribed for other indications than anxiety (for example, SSRIs for depression). The uptake of psychotherapy among patients could not be studied, because it is not registered in the database used.

### Comparison with existing literature

#### Trends in the prevalence and incidence of anxiety.

The recent increase in the registration of anxiety in this study corresponds to the international literature. Although methodologically different, recent increases have been seen in young people across multiple studies in different countries.^24^^–^26 This may be because of actual disease burden or an increased acceptance and awareness of mental disorders in general, leading to earlier recognition. Some studies point in the direction of the latter.^27^^,^^28^ Other authors blame the emergence and importance of social media in the lives of young adults.^29^

From the data in the current study it is unclear whether the increase in the registration of anxiety is actual or perceived. Of note is that the current study used the ICPC-2 code P74, which is a clinical diagnosis. GPs might be hesitant to diagnose a disorder based solely on subclinical symptoms, which is why in some studies there seems to be a recent tendency to code anxiety symptoms rather than diagnoses.^25^^,^^30^ This might also explain why the registered prevalence and incidence of anxiety in the current study are decidedly lower than in other similar studies.^14^^,^^25^^,^^31^ Furthermore, as stated in a previous article by the same team, this increase might also partly be brought about by a registration effect.^19^ This is a form of bias: anxiety could be more likely to be registered now than earlier in the study period for various reasons (for example, more diligent and more frequent coding and registration).

The final 2 years of the study period were marked by the ongoing COVID-19 pandemic, during which mental health disorders, particularly anxiety, seemed on the increase.^32^^,^^33^ The current results show an increasing incidence and prevalence for anxiety in those years, but a COVID-19-specific analysis was not performed. The Belgian public health institute Sciensano found that self-reported mental health in Belgium fluctuated during the pandemic depending on the specific phase (restrictions versus relaxation of measures).^34^ It is conceivable that many patients did not seek help for anxiety during the first waves of the pandemic, making figures in the current study likely underestimates.

For the calculations in the current study it was assumed anxiety disorder was chronic, a stance shared by other authors as well.^35^ A sensitivity analysis was also conducted using a 1-year contact-free interval and the conclusions remain robust. Using this much shorter period a lower prevalence was arrived at, which is mathematically evident (see Supplementary Table S2). The trends were largely conserved (see Supplementary Figure S2).

#### Trends in comorbidities.

The most salient finding here was the fact that the average number of chronic diseases for each patient increased significantly. This is in line with previous findings from the same author group concerning depression.^19^ Patients presenting with mental health disorders in primary care tend to become more complex over time. However, it is also possible that GPs’ awareness of anxiety might be increased specifically in patients with comorbidities.

In any case, a multidisciplinary approach to these patients is warranted. The GP is perfectly placed to coordinate care between first- and second-line health professionals. In an analysis by Martín-Merino *et al*, patients with anxiety were more likely to misuse alcohol, have chronic obstructive pulmonary disease (COPD) or cancer, or be admitted to hospital or referred for another health problem.^31^ In the current sample, there was no concurrent trend for alcohol misuse, although anxiety was significantly associated with a number of somatic conditions, such as asthma and COPD, cardiovascular problems, and cancer.

The strong association with depression is not new and controversy exists as to whether the presence of anxiety might predict the onset of depression or vice versa,^15^ or even whether they constitute different syndromes altogether.^13^^,^^14^

#### Trends in medication.

In the prevalent anxiety case group, the relative amount of psychoactive medication prescriptions increased, albeit only significantly so for the final 6 years. This is consistent with a study by Noordam *et al*, who also found an increase in prescriptions for mental health disorders.^36^ Female patients with anxiety in the current study received proportionally more prescriptions than males, which was the case in the Noordam *et al,* study as well.^36^ Chronic use, which was defined in the current study as ≥3 prescriptions per year, increased significantly, whereas occasional use (one prescription per year) declined. For benzodiazepines specifically, the definition of chronic use in international literature tends to vary and is rarely uniform across studies.^37^

The most frequently prescribed medications in the current study were SSRIs, anxiolytics, and hypnotics. Initially, anxiolytics were prescribed more than SSRIs but the two switched positions around 2017. SSRIs are also a first-line treatment for depression, and the important comorbidity between anxiety and depression might also have been a driving force. According to the National Institute for Health and Care Excellence guidelines, the recommended treatment for anxiety disorder, besides psychotherapy, is SSRIs followed by serotonin–noradrenaline reuptake inhibitors.^38^ The use of benzodiazepines is explicitly discouraged, except for short-term crisis management. This might also have contributed to the prescription trends seen in the data in the current study.

Another interesting trend is the recent steep increase of prescriptions (such as trazodone) in the neuromodulator group.

### Implications for practice

In this registry-based study, a significantly increasing prevalence and incidence of physician-registered anxiety was found, particularly in the past 5 years and in the youngest age groups. Patients with anxiety tend to become more complex or patients with multimorbidity are at least more easily diagnosed with anxiety. A holistic, multidisciplinary primary care approach for these patients, with the GP taking on a coordinating role, is important.

Without knowing about referrals for psychotherapy, treatment for anxiety in Belgian primary care seems to be heavily dependent on medication. In any case, there has been more adherence to official guidelines recently with the use of SSRIs increasing and the use of anxiolytics declining.

## Data

The dataset can be made available subsequent to further de-identification on reasonable request to the corresponding author.
